# Correction: Dosage suppressors of *gpn2*^*ts*^ mutants and functional insights into the role of Gpn2 in budding yeast

**DOI:** 10.1371/journal.pone.0344594

**Published:** 2026-03-09

**Authors:** Le Wang, Pan Li, Pei Zeng, Debao Xie, Mengdi Gao, Lujie Ma, Aamir Sohail, Fanli Zeng

There are errors in the Funding statement. The correct Funding statement is as follows: This work was supported by grants from the National Key Research and Development Program of China (2023YFD1401500 to F.Z.), the National Natural Science Foundation of China (no. 32070574 to F.Z.), the Hebei Provincial Science Foundation for Outstanding Young Scholars (C2023204155 to F.Z.), and Operation Expenses for Universities’ Basic Scientific Research of Hebei Province (KY2024043 to F.Z.).
