# Supplementary figures and images for: Dosage suppressors of gpn2ts mutants and functional insights into the role of Gpn2 in budding yeast
Source: PLoS One. 2024 Dec 6;19(12):e0313597. doi: 10.1371/journal.pone.0313597 (PMC11623451; doi:10.1371/journal.pone.0313597)

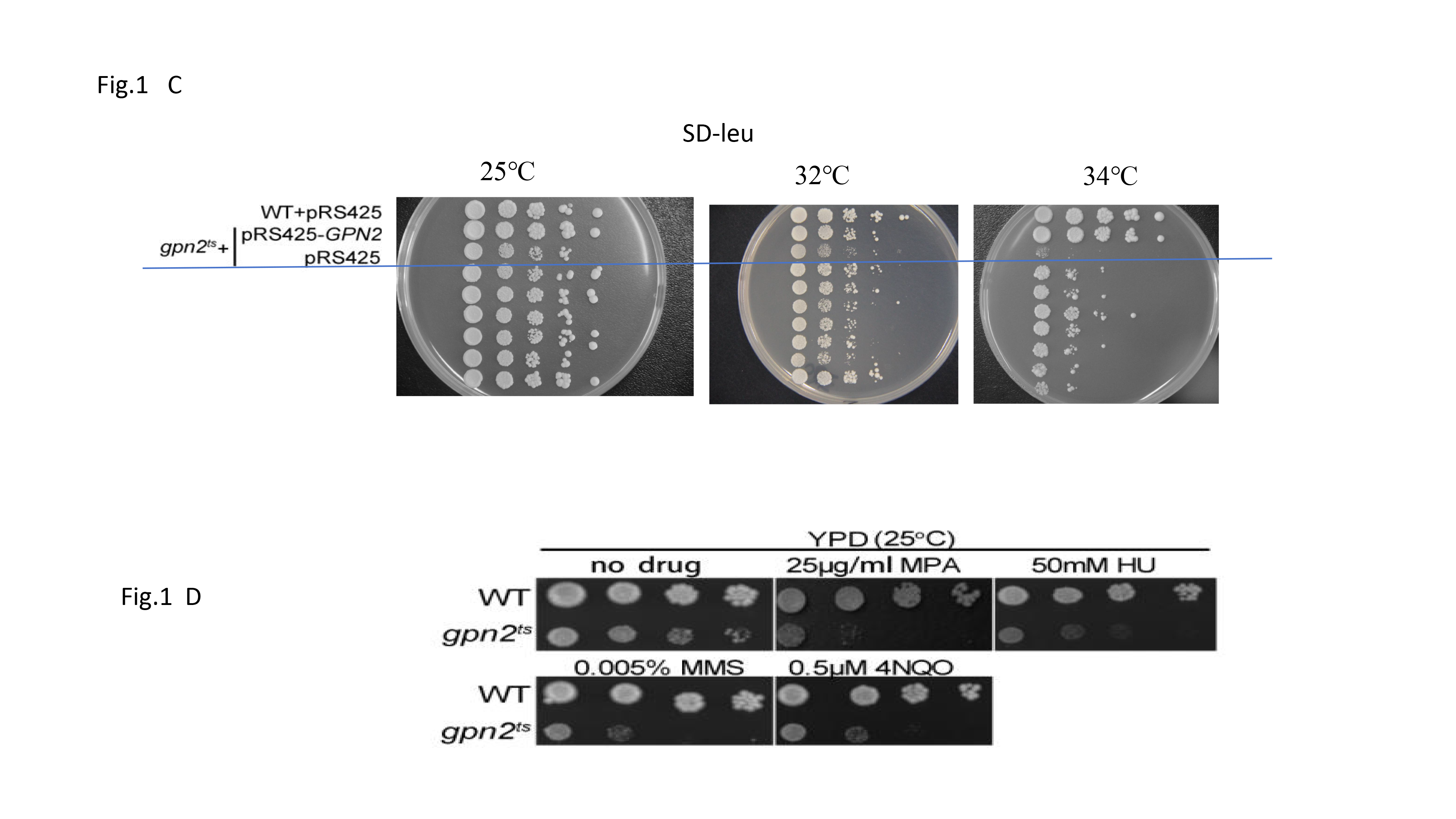

Supplement: S1 Raw data — (ZIP) [file pone.0313597.s001.zip › Raw data/Fig 1 .tif]
