# Supplementary material for: Dosage suppressors of gpn2ts mutants and functional insights into the role of Gpn2 in budding yeast
Source: PLoS One. 2024 Dec 6;19(12):e0313597. doi: 10.1371/journal.pone.0313597 (PMC11623451; doi:10.1371/journal.pone.0313597)
Supplement: Attachment — Submitted filename: Response to Reviewers.docx [file pone.0313597.s002.docx]

Dear Editor,

Thank you for providing us with the opportunity to resubmit our manuscript. We are delighted to know that it has been received positively by the reviewers and is provisionally accepted pending successful revision. Please find attached our revised manuscript along with the updated figures. We have made every effort to fully address the critiques provided, which are outlined in our detailed responses to the reviewers.

Reviewer #1: The Gpn family members are a fascinating yet poorly understood family of proteins and a suppression screen is a valid approach to enhance our knowledge about Gpn2 function. The technical aspects of this submission are fine, using standard methods for genetic suppression screening and validation.

Comments-1: Figure 1A - this domain should read GTP binding, not ATP binding as written.

Response 1: Thank you for your suggestion. We have revised “ATP” to “GTP” in Figure 1A.

Comments-2: Figure 3 contains several spot assays to confirm that genetic suppression of the *gpn2* mutant is occurring. However, some genes described in the results section such as *MRPL27* and *RFC1* have little to no effect on growth. These should be removed from the list of positive suppressors or growth curve data should be generated to confirm that there is improved growth due to expression of these marginal suppressors.

Response 2: Thank you for your suggestion. Some genes described in the results section, such as *MRPL27* and *RFC1*, exhibit minimal or no effect on growth at 34°C. However, they can compensate growth defects of the *gpn2^ts^* mutants at 32°C. Therefore, we consider these genes as candidate suppressors, as detailed in Table 1.

Comments-3: This read like a list making exercise and many of the descriptions of protein function listed in the results section are taken verbatim from the Yeast Genome Database pages for these genes which borders on plagarism.

Response 3: Thank you for pointing this out. We have significantly revised the manuscript to include additional research and our original insights. Please refer to L156-L176, L178-190, L195-L215, L217-L227, L229-L245, L247-L273.

Comments-4: I would have liked to see some attempt to further investigate how these suppressors affect growth of the *gpn2* mutant cells. An easy experiment would be to look at RNAPII subunit localization with a GFP tagged protein as these are readily available in the yeast community.

Response 4: Your suggestions have been invaluable. We tagged Rpb1, the largest subunit of RNA polymerase II, with GFP in the *gpn2^ts^* mutant and subsequently overexpressed it in high-copy suppressors *PAB1*, *CDC5*, and *RGS2* at 34°C. We found that *CDC5* significantly compensated for the nuclear dispersion observed in the mutant (Figure 5C, L329-338).

Comments-5: The *RGS2* suppressor result is interesting and should have been followed up upon. Perhaps transforming the *RGS2* plasmid into *gpn1* or *gpn3* mutants to assess whether it's GAP activity restores growth of these mutants at elevated temperatures.

Response 5: Thank you for your valuable suggestion. We have transformed the high-expression constructs of *PAB1*, *CDC5*, and *RGS2* into the *gpn1^ts^* and *gpn2^ts^* mutants and cultured them at various temperatures. Our findings indicate that they can alleviate the growth defects of *gpn2^ts^* in the presence of DNA-damaging and transcriptional suppressors drugs at 32°C. Additionally, at 34°C, *PAB1*, *CDC5*, and *RGS2* each significantly reduced the sensitivity of *gpn1^ts^* mutants on 4-NQO tablets, with *CDC5* showing a particularly notable effect (Figure 5A and B, L314-323)

Comments-6: There are considerable issues with phrasing, economical use of words and grammar and the authors would benefit from having someone with strong written English skills review their writing before submission.

Response 6: Thank you for your suggestions. We have thoroughly addressed the issues raised and have made comprehensive revisions to the language of our manuscript. To ensure accuracy in word choice, grammar, and sentence structure, the revised manuscript has been meticulously edited by an experienced science editor from A&L Scientific Editing ([www.alpublish.com](http://www.alpublish.com/)), whose first language is English and who specializes in editing scientific papers authored by non-native English speakers. We sincerely hope that the revised manuscript meets your expectations. We are grateful for your consideration of our work and would like to express our heartfelt appreciation once again.

Reviewer #2:

Comments-1: First, the authors previously published a similar genetic suppression screen on the *gpn2^ts^* mutant, where they described the suppressors Rpb12, Rba50, and other GPNs (Zeng et al, 2018). Therefore, they should clarify whether this study is a continuation of that one. If so, they should name the *RPB12* and *RBA50* genes as suppressors, indicating that their function has been previously studied. If the study presented in this manuscript is a different screen, the authors should clarify why they do not find *RPB12* and *RBA50* as suppressors in this case.

Response 1: Thank you for your suggestion. This study builds upon previous research. Our research group has already described the suppressors Rba50, Rpb12, and other GPNs in a prior study (Zeng et al, 2018), as noted in the manuscript (L66-69). In addition, this study aims to identify novel inhibitors, thereby providing a more robust theoretical foundation for investigating the role of Gpn2 in RNA polymerase II activity.

Response 2: Similarly, the sensitivity of the *gpn2^ts^* mutant to mycophenolic acid had been previously published and should therefore be indicated in the text.

Response 2: Thank you for your suggestion. We have documented the sensitivity of the *gpn2^ts^* mutant to mycophenolic acid in the revised manuscript (L44-45).

Response 3: The authors should clarify why they analyse the sensitivity of *gpn2^ts^* mutant to the other drugs but then do not use them to check the suppressors. They only look the suppression of three plasmids in DNA-damaging agents. They should look the suppression in the presence of all the studied drugs, as the effect of different drugs might vary depending on the suppressor gene, which could help clarify the function that the gene is correcting.

Response 3: Thank you for your valuable suggestions. In response, we have added experiments to assess the sensitivity of the transcriptional suppressor mycophenolic acid (MPA) to *gpn1^ts^* and *gpn2^ts^* strains. This further elucidates that the suppressors *PAB1*, *CDC5*, and *RGS2* are effective not only against DNA-damaging drugs but also at the transcriptional level. *CDC5* exhibited the most pronounced inhibitory effect. Please refer to Figures 5A and B. Details are provided in the manuscript (L314-325).

Comments-4: As minor corrections, in the third paragraph of the introduction, the authors write “interaction between Rpb12, Rpb3, and Rba50 subunits.” However, Rba50 is not a subunit of RNA pol II but an associated factor, so the wording should be corrected.

Response 4: Thank you for your suggestion. We have corrected this in our manuscript (L41-42).

Comments-5: The beginning of the third paragraph of the introduction is confusing and should be rewritten: “In the preceding work conducted, it was found that Gpn2, one of the vital factors in RNA polymerase II assembly, is directly participating in the interaction between Rpb12, Rpb3, and Rba50 subunits. On the contrary, genetic screening data also confirmed the association of Gpn2 with Rba50 and Rpb3, which are the second-largest subunits of RNA polymerase II assembly factors.”

Response 5: Thank you for pointing out the confusion. We have revised the beginning of the third paragraph of the introduction for clarity (L40-43).
