# Supplementary material for: Dosage suppressors of gpn2ts mutants and functional insights into the role of Gpn2 in budding yeast
Source: PLoS One. 2024 Dec 6;19(12):e0313597. doi: 10.1371/journal.pone.0313597 (PMC11623451; doi:10.1371/journal.pone.0313597)
Supplement: Attachment — Submitted filename: Response to Reviewers.docx [file pone.0313597.s003.docx]

Dear Editor,

We are very pleased to know that our manuscript was received positively by the reviewers and that it is provisionally accepted, pending successful minor revision. Please find attached our revised manuscript. We have made every effort to fully address the critiques, which are outlined in our replies to reviewers. Please note the itemized responses below, where the original comments are underlined in black and our responses are displayed in blue. We sincerely hope that the updated version meets your expectations. We are grateful for your consideration of our publication and would like to express our heartfelt appreciation once more.

Reviewer #1:

Figure 2 and 4 not included in the resubmission. I am assuming there were no changes from the original manuscript.

Figure 5. I would remove the wording "significantly" from the statement about *CDC5* overexpression restoring Rpb1 localization in the *gpn2* mutant as there was no quantitative analysis done. However, the qualitative result is fairly obvious.

Methods section should include a line about Rpb1-GFP whether it was plasmid based or integrated into the genome.

Comments-1: Thank you very much for your valuable feedback. Figures 2 and 4 were no changes from the original manuscript. We have thoroughly reviewed and revised the entire manuscript, removing the wording "significantly" from the statement about *CDC5* overexpression restoring Rpb1 localization in the *gpn2* mutant. Additionally, we have included a description of Rpb1-GFP in the Materials and Methods section (L88-91).
